# Supplementary material for: What Is the Relationship Between Efficacy of Seed Treatment with Insecticides Against Dalbulus maidis (Delong and Wolcott) (Hemiptera: Cicadellidae) Healthy and Infected with Spiroplasm in the Corn Stunt Control?
Source: Insects. 2025 Jul 11;16(7):713. doi: 10.3390/insects16070713 (PMC12295506; doi:10.3390/insects16070713)
Supplement: Supplementary file 1 [file insects-16-00713-s001.zip › insects-3700664-supplementary.pdf]

insects

MDPI

Supplementary Materials: Table S1. Rank of leafhopper daily mortality (%) after different insecticide treatment during five days in 0, 5, 10, 15 and 20 days after infestation.

	**Imidacloprid/ Tiodicarb**		**λ- cialotrin/ Thiametoxam**		**Imidacloprid**		**Clothinidin**		**Thiametoxam**		**T. methyl + Fipronil/ Thiametoxam**	
**Hours**	Infective	Health	Infective	Health	Infective	Health	Infective	Health	Infective	Health	Infective	Health
0 days after the appearance of the second expanded leaf												
**24**	85.7 Aa	85.7 Aa	28.6 Bc*	0 Cb	85.7 Aa*	14.3 Bb	28.6 Cc*	0 Bb	57.1 Bb*	0 Bb	71.4 Aab*	0 Bb
**48**	85.7 Aa	85.7 Aa	42.9 Bb	28.57 Bb	85.7 Aa*	28.6 Bb	42.9 BCb*	0 Bc	71.4 ABa	0 Bc	85.7 Aa	0 Bc
**72**	85.7 Aa	100 Aa	71.4 Aa*	42.86 Bb	85.7 Aa*	42.9 ABb	57.1 Bb*	14.3 Bc	85.7 Aa*	0 Bd	85. Aa*	0 Bd
**96**	85.7 Aa	100 Aa	71.4 Aa	71.43 Aa	100 Aa*	57.1 Ab	57.1 Bb*	14.3 Bc	85.7 Aa*	14.3 Bc	85.71 Aa*	0 Bd
**120**	85.7 Aa	100 Aa	71.4 Aa	71.43 Aa	100 Aa*	57.1 Ab	71.4 Aa*	42.9Ab	100 Aa*	42.9 Ab	100 Aa*	42.9 Ab
**5** days after the appearance of the second expanded leaf												
**24**	14. Bc	0 Ba	0 Bd	14.29 Ba	100 Aa*	14.29 Ca	57.14 Bb*	0 Ba	28.57 Cc*	0 Ba	0 Cd	0 Ca
**48**	14.29 Bc	0 Ba	14.29 Bc	14.29 Ba	100 Aa*	14.29 Ca	57.14 Bb*	0 Ba	57.14 Bb*	0 Ba	57.14 Bb*	0 Ca
**72**	14.29 Bb	14.29 ABb	14.29 Bb	28.57 Bab	100 Aa*	42.86 Ba	100 Aa*	0 Bb	100 Aa*	14.29 Bb	100 Aa*	14.29 Cb
**96**	57.14 Ab*	14.29 ABc	42.86 Ab	71.43 Aab*	100 Aa	85.71 Aa	100 Aa*	14.29 Bc	100 Aa*	14.29 Bc	100 Aa*	57.1 Bb
**120**	57.14 Ab*	28.57 Ac	57.14 Ab	85.71 Aa*	100 Aa	85.71 Aa	100 Aa*	42.86 Ab	100 Aa*	57.14 Aab	100 Aa	71.43 Aa
**10** days after the appearance of the second expanded leaf												
**24h**	14.3 Ca	0 Ba	0 Ba	0 Ba	14.29 Ca	0 Ba	14.29 Ba	0 Ba	14.29 Ca	0 Ba	14.29 Ba	0 Ba
**48h**	57.1 Bab*	28.6 Aa	42.9 Ab*	28.6 Aa	42.9 Bb*	28.6 Aa	71.4 Aa*	28.6 Aa	57.1 Bab*	28.6 Aa	71.4 Aa*	14.3 ABa
**72h**	71.4 Aa*	28.6 Aa	57.1 Ab	42.9 Aa	85.7 Aa*	28.6 Aa	71.4 Aa*	28.6 Aa	85.7 Aa*	28.6 Aa	71.4 Aa*	28.6 Aa
**96h**	71.4 Aa*	28.6 Aa	57.1 Ab	42.9 Aa	85.7 Aa*	28.6 Aa	85.7 Aa*	28.6 Aa	85.7 Aa*	28.6 Aa	100 Aa*	28.6 Aa
**120h**	71.4 Aab*	42.9 Aa	57.1 Ab	42.9 Aa	85.7 Aa*	28.6 Aa	85.7 Aa*	28.6 Aa	85.7 Aa*	28.6 Aa	100 Aa*	28.6 Aa
**15** days after the appearance of the second expanded leaf												
**24**	57.1Bb*	0 Aa	57.1 Bb*	0 Aa	71.4 Aa*	0 Ba	71.4 Aa*	14.3 Aa	28.6 Bc*	0 Aa	28.6 Cc*	0 Aa
**48**	71.4 Aa*	0 Aa	57.1 Bb*	0 Aa	71.4 Aa*	14.3 Ba	71.4 Aa*	14.3 Aa	42.9 Ab*	0 Aa	42.9 Bb*	0 Aa
**72**	71.4 Aa*	14.2 Ab	100 Aa*	0 Ab	71.4 Aa*	42.9 Aa	71.4 Aa*	14.3 Ab	42.9 Ab*	0 Ab	42.9 Bb*	0 Ab
**96**	71.4Aa*	14.29 Ab	100 Aa*	0 Ab	85.71 Aa*	42.86 Aa	71.43 Aa*	14.29 Ab	42.86 Ab*	14.29 Ab	57.14 Bb*	0 Ab
**120**	71.4 Aa*	14.3 Ab	100 Aa*	14.3 Ab	100 Aa*	42.9 Aa	71.4 Aa*	14.3 Ab	57.1 Ab*	14.3 Ab	71.4 Aa*	14.29 Ab
**20** days after the appearance of the second expanded leaf												
**24**	0 Ba	0 Aa	0 Aa	0 Ba	0 Ba	0 Ba	0 Ca	0 Aa	0 Ba	0 Aa	0 Ba	0 Aa
**48**	14.3 Bb	0 Aa	0 Ab	0 Ba	42.9 Aa*	0 Ba	14.3 BCb	0 Aa	14.3 Bb	0 Aa	14.3 Bb	0 Aa
**72**	14.3 Bb	0 Ab	14.3 Ab	0 Bb	57.1 Aa*	28.6 Aa	28.6 Bb*	0 Ab	42.9 Aab*	14.3 Aab	14.3 Bb	14.3 Aab
**96**	42.9 Aa*	0 Ab	14.3 Ab	0 Bb	57.1 Aa*	28.6 Aa	42.9 Aa*	14.3 Aab	42.9 Aa*	14.3 Aab	42.9 Aa*	14.3 Aab
**120**	42.9 Aa*	14.3 Aa	14.3 Ab	28.6 Aa	57.1 Aa*	28.6 Aa	42.9 Aa*	14.3 Aa	42.9 Aa*	14.3 Aa	42.9 Aa*	14.3 Aa

Different capital letters in the same column means statistical differences in mortality within different time of evaluation (24h, 48h, 72h, 96h and 120h). Different lowercase letters in the same line means statistical differences in mortality within different insecticide treatment in the same group (infective or healthy). * means statistical differences in mortality between infective and healthy leafhopper within the same insecticide treatment and within the same time of evaluation.

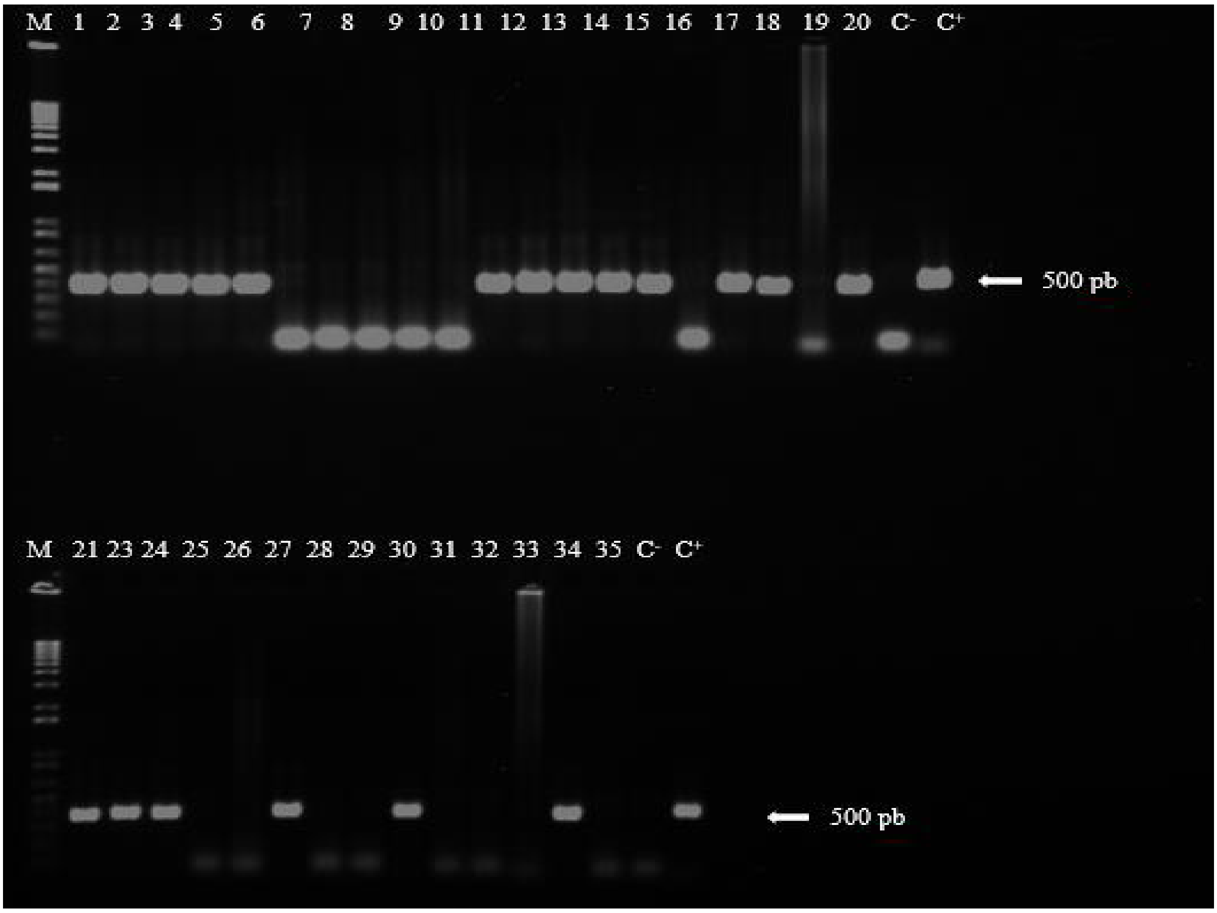

Figure S1. Polymerase chain reaction (PCR) at time 0. Each number represents a treatment replicate. The samples 1-5 (=Control), 6-10= Imidacloprid/Thiodicarb, 11-15= Lambda-Cyhalothrin/Thiamethoxam, 16-20= Imidacloprid, 21-25= Clothianidin, 26-30= Thiamethoxam, and 31-35= Thiofanate-Methyl + Fipronil/Thiamethoxam. (M: 1 kb plus DNA ladder (Invitrogen®); C+: positive control (Spiroplasma) and C.−: negative control (all components of the PCR reaction except DNA).
